# Management of Low Back Pain With Concurrent Hamstring Tightness: A Case Report Highlighting the Efficacy of Proprioceptive Neuromuscular Facilitation, Mulligan’s Two-Leg Rotation Technique, and Exercise Regimen

**DOI:** 10.7759/cureus.58705

**Published:** 2024-04-22

**Authors:** Priya Tikhile, Deepali S Patil, Pratik R Jaiswal

**Affiliations:** 1 Musculoskeletal Physiotherapy, Ravi Nair Physiotherapy College, Datta Meghe Institute of Higher Education & Research, Wardha, IND; 2 Sports Physiotherapy, Ravi Nair Physiotherapy College, Datta Meghe Institute of Higher Education & Research, Wardha, IND

**Keywords:** mulligan’s two-leg rotation, rehabilitation, motor control exercise, proprioceptive neuromuscular facilitation, low back pain

## Abstract

Low back pain (LBP) is a prevalent musculoskeletal issue characterized by discomfort in the lumbosacral region. LBP localized between the 12th thoracic vertebra and inferior gluteal folds is common and often lacks a clear etiology. Various factors contribute to LBP, including increased lumbar lordosis, diminished abdominal muscle strength, reduced back extensor muscle endurance, and flexibility limitations in the back extensors, iliopsoas, and hamstrings. Treatment modalities for LBP encompass surgical intervention, pharmacotherapy, lumbar injections, psychotherapy, chiropractic care, and physiotherapy, with manual therapy being a prominent approach. Physiotherapists employ a spectrum of manual techniques, including mobilization, manipulation, and massage, to address LBP. Hamstring flexibility plays a pivotal role in spinal mechanics, and tight or shortened hamstrings can exacerbate LBP. Mulligan's techniques, notably the two-leg rotation (TLR) technique, are valuable interventions for addressing hamstring tightness in cases of LBP. Proprioceptive neuromuscular facilitation (PNF) was also used to manage pain and improve strength. This case report outlines the management of a 32-year-old male presenting with LBP and hamstring tightness coupled with core muscle weakness. Through comprehensive assessment and physiotherapeutic interventions, significant improvements were observed in pain intensity, lumbar range of motion, disability, straight leg raise (SLR), and core muscle strength following a three-week physiotherapy intervention.

## Introduction

Factors contributing to the development of low back pain (LBP) include increased lumbar lordosis, hampered abdominal muscle strength, reduced back extensor muscle endurance and flexibility, shortened iliopsoas and hamstring muscles, alterations in body composition, and other related factors. Additionally, reduced spinal mobility due to disruptions in muscle synergies can increase the energy expenditure of maintaining posture [1]. Chronic primary LBP is defined by the World Health Organization as persistent or recurrent pain lasting more than three months without a clear underlying disease process, structural lesion, or deformity. Chronic low back pain (CLBP) predominates, affecting approximately one in six adults globally but can often be effectively managed without surgery in primary and community care settings [2]. Pain stemming from the lumbar, lumbosacral, or sacroiliac regions varies depending on the affected area. The classification of LBP depends on its cause, distinguishing between specific and non-specific categories. LBP can manifest as acute (lasting 0-14 days), sub-acute (lasting two to 12 weeks), or chronic (persisting for more than three months) based on its duration [3]. Non-specific low back pain (NSLBP) lacks a clear relationship between symptoms, physical examination findings, and imaging results. Chronic non-specific low back pain (CNLBP) often arises from physiological structural fragility in the lumbar region and can be exacerbated by poor posture, resulting in functional damage. NSLBP primarily correlates with posture or improper body mechanics, along with various anatomical issues contributing to the condition [1]. Risk factors for NSLBP include poor hamstring flexibility, inadequate abdominal strength, heightened levels of physical activity, and work-related postural stress. The hamstring muscle, being biarticular, is prone to shortening even under normal circumstances, given its role as a postural muscle. This superficial two-joint muscle has a propensity to tighten, leading to muscle imbalances and potential postural issues, increasing susceptibility to injury. Limited activity and a lack of regular exercise contribute to the high prevalence and incidence of hamstring tightness in individuals with NSLBP [4].

The hamstring muscle group, comprising the semitendinosus, semimembranosus, and biceps femoris muscles, is frequently impacted by excessive stress. Prolonged periods of sitting can further diminish hamstring mobility, exacerbating the risk of discomfort and injury [5]. Tight hamstrings with weak core muscles restrict mobility of the pelvis, potentially leading to alterations in lumbar pelvic rhythm. These changes in biomechanics can increase strain on the lumbar segment, contributing to the development or exacerbation of LBP [6]. Various treatment modalities are available for managing LBP, including surgery, oral medication, lumbar region injections, psychotherapy, chiropractic care, and physiotherapy. Manual therapy, a common approach in physiotherapy, encompasses a wide array of techniques such as mobilization, manipulation, core strengthening exercises, motor control exercises, and electrotherapy, among others [7]. Dysfunction in activating and coordinating trunk muscles can lead to excessive movement and instability in the lower back, potentially exacerbating pain and limiting function [8].

Proprioceptive neuromuscular facilitation (PNF) is a therapeutic approach that enhances proprioception by stimulating neuromuscular responses, which can improve pain management, muscle strength, range of motion, and functional mobility in individuals with skeletal muscle weakness [9]. Mulligan's approach to manual therapy emphasizes the restoration of normal joint arthrokinematics and osteokinematics through mobilization with movement. Unlike traditional stationary mobilization techniques, Mulligan recognizes the need for dynamic movements to address positional faults arising from injury or prolonged use. By integrating mobilization with movement, Mulligan's concept aims to alleviate pain and improve the range of motion by correcting postural faults within the joint [10]. Mulligan’s two-leg rotation (TLR) technique offers a painless approach applicable to patients experiencing hamstring tightness, LBP, or limited and/or painful straight leg raise (SLR). TLR presents a valuable option, particularly beneficial for individuals with significant bilateral restrictions in straight leg raising [4].

The case study delves into the experience of a 32-year-old office worker who sought physical therapy upon receiving a diagnosis of LBP accompanied by hamstring tightness. Throughout the course of treatment, the implementation of PNF and Mulligan’s mobilization, alongside other therapeutic interventions, played a pivotal role in not only alleviating pain but also in bolstering core strength and enhancing hamstring muscle flexibility. The exercises described in our case report are intended for patients with LBP, including both specific CLBP and non-specific CLBP. While the treatment approach may vary based on individual patient assessments and needs, the exercises outlined aim to address common underlying issues such as hamstring tightness, core muscle weakness, and impaired lumbar mobility, which are often present in both specific and non-specific forms of CLBP. It is important to note that the exercises detailed in the case report are not universally applicable to all patients with LBP. Rather, they should be tailored to each individual's condition, considering factors such as the underlying cause of the pain, severity of symptoms, and any coexisting medical conditions.

## Case presentation

Patient information

A 32-year-old male, employed as an office worker, presented to the physiotherapy department with complaints of LBP. The patient reported experiencing CLBP for the past five months, with symptoms being milder in the morning and worsening with work. His occupation involved prolonged periods of sitting, with a history of heavy lifting. The pain had a gradual onset and progressively worsened, particularly aggravated by bending and daily activities. Previous consultation with an orthopedician resulted in prescribed medications providing temporary relief. However, a recent exacerbation of pain prompted a visit to an orthopedic surgeon, where clinical examination revealed paraspinal region spasm and identified hamstring muscle tightness. Despite pain medications, the symptoms persisted, leading to referral to the physiotherapy department. The patient was subsequently recommended for physiotherapy rehabilitation aimed at pain reduction, increasing range of motion, alleviating muscle tightness, restoring full mobility, and enhancing overall quality of life.

Clinical findings

On examination, the patient was conscious and oriented. Before beginning the examination, the patient's consent was obtained. A spinal examination revealed no anomalies. Further examination reveals a positive straight leg raise (SLR) at 45° on the right side, indicating hamstring tightness. Tender was present along the paraspinal area. The individual described a persistent dull ache rating it as 5/10 at rest and 8/10 during activity on the visual analogue scale. According to the modified Oswestry disability index (MODQ), the patient experienced a 58% impairment. Evaluation with the modified Schober test revealed 4.4 cm of lumbar flexion. Radiological examinations indicated normal vertebral and disc dimensions, as well as proper bone alignment. The slump test yielded negative results. The pre- and post-physiotherapy intervention values of SLR, pain measured on the visual analog scale, and disability by modified Oswestry disability are given in Table 1. The range of motion of lumbar flexion and extension and side (lateral) lumbar flexion is in Table 2 and lumbar manual muscle testing is in Table 3.

**Table 1:** Pre- and post-physiotherapy intervention outcome measures SLR: straight leg raise; VAS: visual analog scale; MODQ: modified Oswestry disability index

Outcome measure	1st week		3rd week
	Pre-intervention	Post-intervention	Post-intervention
SLR	45*°*	50^0^	70^0^
VAS	5/10 on rest; 8/10 on activity	3/10 on rest; 5.5/10 on activity	0/10 on rest; 1/10 on activity
MODQ	58%	45%	23%

**Table 2:** Pre- and post-physiotherapy intervention range of motion by modified Schober test

Joint	1st week		3rd week
	Pre-intervention	Post-intervention	Post-intervention
Lumbar flexion	4.4 cm	5.2 cm	6.3 cm
Lumbar extension	3 cm	5 cm	8 cm
Side (lateral) lumbar flexion	Right: 2 cm	Right: 3 cm	Right: 5 cm
	Left: 2 cm	Left: 3 cm	Left: 5 cm

**Table 3:** Lumbar manual muscle testing

Muscles	Grades		
	1st week		3rd week
	Pre-intervention	Post-intervention	Post-intervention
Lumbar flexors	3/5	4/5	4+/5
Lumbar extensors	3/5	4/5	4+/5
Side(lateral) lumbar flexors	3/5	4/5	4+/5
Lumbar spine rotators	3/5	4/5	4+/5

Therapeutic intervention

**Figure 1:**
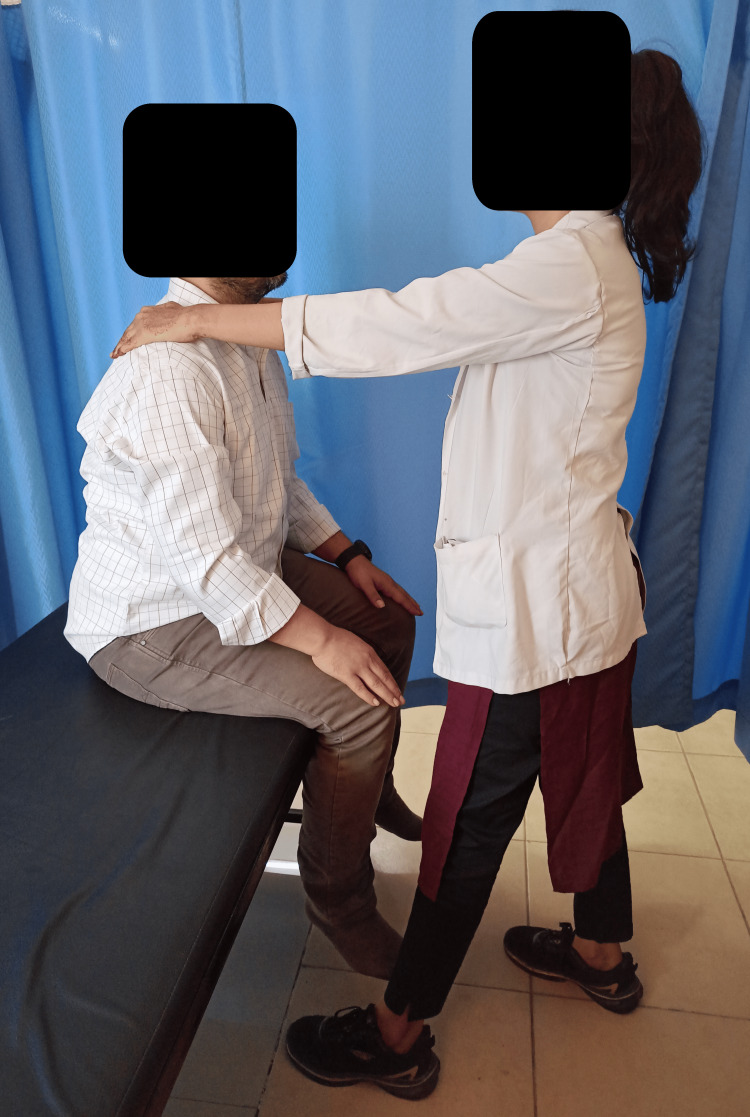
IC of trunk extensors IC: isometric contractions

**Figure 2:**
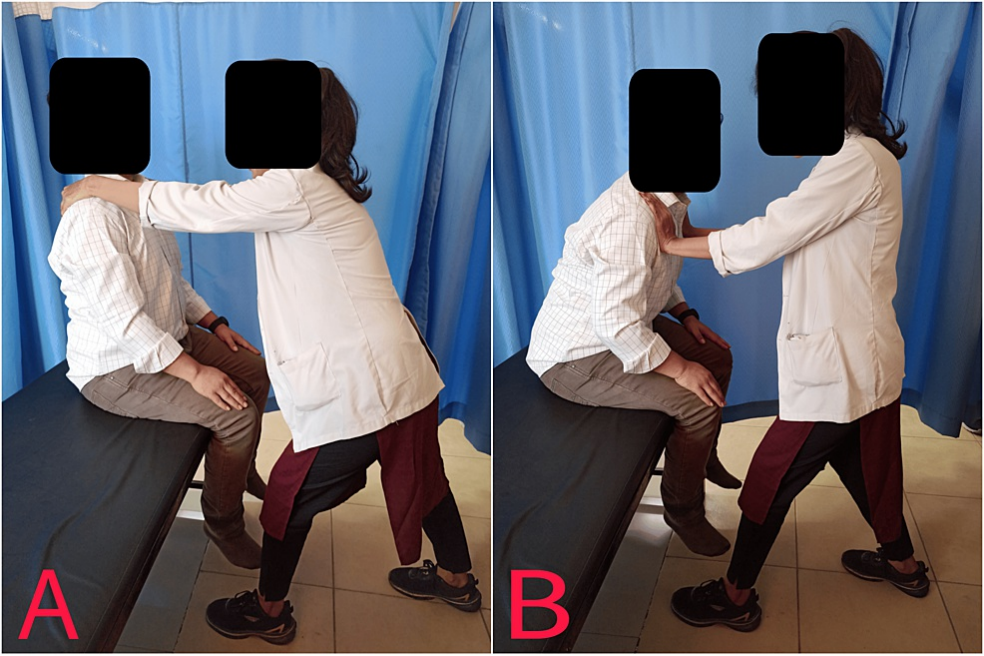
Alternating EC (A) and CC (B) of the trunk flexors EC: eccentric contractions; CC: concentric contraction

**Figure 3:**
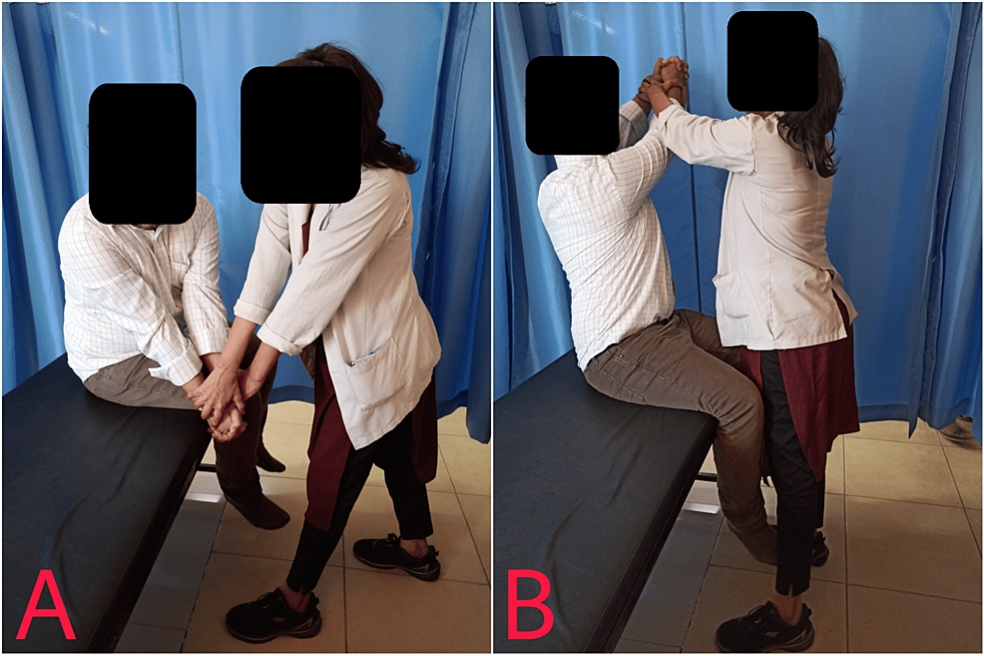
Alternate chop and lift movement patterns. Starting position (A) and ending position (B)

**Figure 4:**
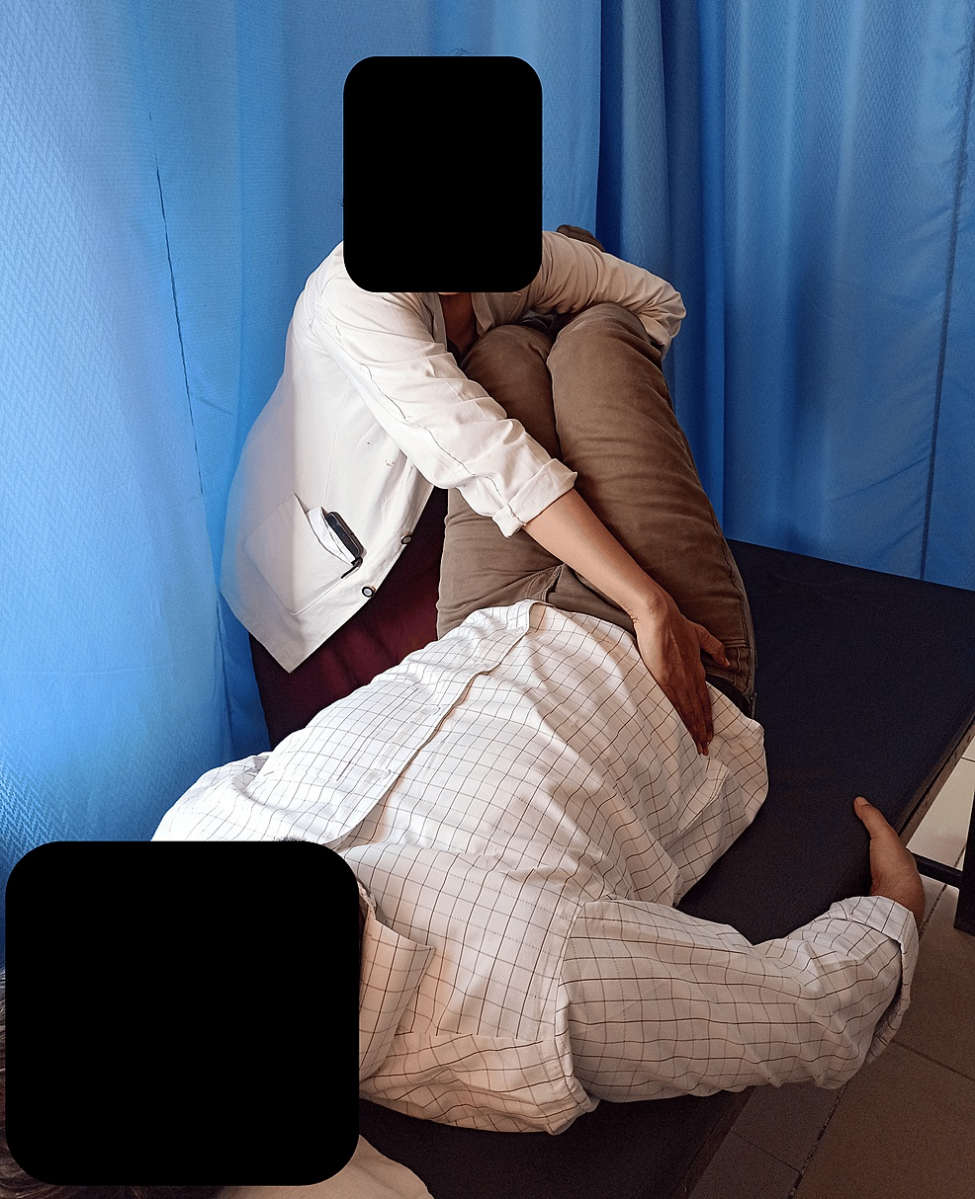
Mulligan's TLR TLR: two-leg rotation

The objectives of the intervention for managing LBP included alleviating pain, improving hamstring flexibility, and strengthening and mitigating symptom recurrence. Exercise intensity was elevated considering the patient's tolerance. Consistency in performing exercises is vital for enhancing muscle strength and effectively managing LBP. The exercise regimen was systematically advanced and customized to align with the patient's tolerance levels. Table 4 outlines a comprehensive, goal-driven physical therapy rehabilitation program for LBP. Figures 1, 2, 3 illustrate PNF and Figure 4 shows Mulligan's TLR.

**Table 4:** Structured rehabilitation protocol All exercises with 10 seconds hold and 20 repetitions each. IC: isometric contractions; EC: eccentric contractions; CC: concentric contraction; LBP: low back pain; PNF: proprioceptive neuromuscular facilitation; TLR: two-leg rotation

Goals	Therapeutic intervention	Treatment protocol
Patient Education	The patient receives comprehensive information regarding their condition and the significance of engaging in physical rehabilitation	Education was given to the patient about exercises and explaining the do's and don'ts.
To reduce pain	Hot moist pack	For the lower back and hamstring muscles for 10 minutes
	Interferential therapy (four pole vector over the lower back)	For 10 minutes
Strengthening	PNF	Week 1: IC of trunk flexors and extensors (10 s holds). Week 2: Alternating IC and EC of the trunk flexors. 5 s resisted CC of the trunk flexors. 5 s resisted EC of the trunk flexors. Resisted IC of the trunk in neutral and similar for trunk extensors. Week 3: Alternate chop and lift movement patterns in diagonal and spiral directions for 10 s.(maximum resistance provided by the therapist).
To reduce LBP and improve hamstring flexibility	Mulligan’s TLR	The therapist positions themselves beside the supine patient, with both legs flexed to raise the feet off the surface. While ensuring the patient's shoulders remain in contact with the bed, the therapist gently guides the patient's legs to the side. Once the limit of motion is reached, the position is held for 30 seconds while the therapist applies additional pressure. Subsequently, the legs are lowered back to the surface, and this process is repeated for three repetitions, with a one-minute rest. The same method is performed for the opposite extremity.
Boost surrounding muscle strength and increase low back mobility	Motor control exercises	1) Engage the abdominal muscles inward. 2) Combine abdominal contraction with heel slides. 3) Perform leg lifts while drawing in the abdominal muscles. 4) Execute bridging movements while engaging the abdominal muscles. 5) Elevate arms in a quadruped position while maintaining abdominal contraction. 6) Lift the legs in a quadruped position while keeping the abdominal muscles engaged. 7) Alternate lifting arms and legs in a quadruped stance while contracting the abdominals. 8) Side bridge on elbows with knees bent while contracting the abdominals. 9) Side bridge on elbows with knees extended while maintaining contracting abdomen. 10) Perform trunk curls to engage the abdominal muscles.

## Discussion

Hamstring flexibility plays a crucial role in providing mechanical advantage, while tight hamstring can adversely affect spinal mechanics. Restricted pelvic mobility resulting from hamstring tightness and weakened core muscles may lead to lumbar strain. Moreover, tight hamstrings and diminished core muscle strength can compromise the normal lordotic curve, potentially increasing strain on the lumbar spine and disrupting the lumbar pelvic rhythm, contributing to the onset of LBP [11]. CLBP emerges as a significant issue marked by persistent pain, distress, muscular fatigue, disrupted sleep patterns, emotional disturbances, and decreased mobility lasting beyond 12 weeks, significantly impacting daily functioning. Physical exercise presents an opportunity to enhance strength, mobility, endurance, and functional capacity. A range of exercises, including lumbar stabilization exercises, motor control exercises, core strengthening exercises, lumbar flexion exercises, walking exercises, and bracing exercises, have been recommended for managing CLBP. These exercises prioritize lumbar stabilization and core strengthening to alleviate symptoms [12].

Mulligan's techniques have demonstrated effectiveness in enhancing hamstring flexibility, particularly benefiting individuals experiencing significant bilateral limitations in SLR [6]. Mulligan innovatively developed a comprehensive collection of manual therapy techniques, setting them apart from conventional mobilization procedures by integrating movement. These techniques are administered while patients engage in either active or passive movements or during resisted muscle contractions. Mulligan's approach is conducted within the pain-free range of motion, potentially enhancing its safety compared to other manual therapy methods [13].

Gatti et al. suggested that implementing trunk balance exercises and limb and trunk muscle strengthening routines seemed to effectively reduce disability and enhance the physical aspect of quality of life for individuals with CLBP [14]. Hoffman et al. suggest focusing on targeted mechanical restoration through exercise and mobilization in the lumbar region and hips contributes to maintaining a healthy and functional lower back [15]. Hodges et al. emphasized the importance of addressing imbalances in trunk muscles among patients with CLBP through interactive training to promote equilibrium. They highlighted the significance of trunk muscle strength as a foundational element for maintaining spinal stability and overall balance [16]. Kofotolis et al. observed a reduction in lumbar muscle pain among patients with CLBP following PNF [17]. Phansopkar et al. demonstrated in their study that treatment techniques such as Mulligan’s TLR were helpful in enhancing hamstring flexibility among individuals with NSLBP, resulting in improvements in pain levels, range of motion, and functional disability. They underscored the potential of these techniques to be widely utilized in clinical settings to enhance hamstring flexibility [4]. Theologou et al. established through their research that the application of interferential current could contribute to pain reduction and enhance functionality, body posture, walking ability, and balance among patients with CLBP [18]. Vincenzino et al. suggested that the prompt pain-relieving effects of Mulligan’s techniques could be attributed to the activation of non-opioid endogenous pain inhibition pathways. This activation, measured through recordings of sympathetic nervous system activity, particularly involves the descending pain inhibitory systems via the periaqueductal gray regions in the mid-brain [19]. In his randomized placebo-controlled trial, Hidalgo et al. utilized the Oswestry disability index to assess functional improvement. Patients treated with Mulligan’s mobilization with movement reported greater enhancements in outcomes [20].

The integration of traditional physiotherapy with PNF and Mulligan’s TLR technique offers a comprehensive approach to addressing both the symptoms and underlying causes of LBP and hamstring tightness. This combined approach emphasizes a holistic treatment strategy, targeting not only immediate pain relief but also addressing the root issues contributing to the patient's condition. In the case of our patient, the integration of physiotherapy with PNF and Mulligan’s TLR resulted in significant improvements in pain levels, range of motion, and functional disability, consistent with the findings reported in the literature. While no long-term follow-up was conducted in this case report, future studies may explore the long-term effects and sustainability of such interventions. This comprehensive approach, addressing both pain management and functional improvements, ensures a more thorough and enduring recovery process, ultimately promoting the patient's overall health and well-being.

## Conclusions

This case report documents the utilization of physical therapy intervention, specifically employing PNF and Mulligan’s TLR technique along with motor control exercises, to effectively manage CLBP and hamstring tightness. The findings underscore the efficacy of this comprehensive rehabilitation approach by pain reduction, enhanced range of motion, improved lumbar muscle strength, decreased hamstring tightness, and reduced disability. This case contributes significantly to our understanding of successful physiotherapy treatment for individuals with CLBP, particularly those engaged in prolonged periods of sitting with sedentary lifestyles.
